# Landscape heterogeneity drives intra-population niche variation and reproduction in an arctic top predator

**DOI:** 10.1002/ece3.675

**Published:** 2013-07-24

**Authors:** Vincent L'Hérault, Alastair Franke, Nicolas Lecomte, Adam Alogut, Joël Bêty

**Affiliations:** 1 Université du Québec à Rimouski et Centre d'Études Nordiques300 Allée des Ursulines, Rimouski, Quebec, G5L 3A1, Canada; 2 Canadian Circumpolar Institute, University of EdmontonEdmonton, Alberta, Canada; 3 Government of Nunavut Department of EnvironmentP.O. Box 209, Igloolik, Nunavut, Canada; 4 Université de Moncton18 Avenue Antonine-Maillet Moncton, New Brunswick, E1A 3E9, Canada; 5 Nunavut Arctic CollegeP.O. Box 187, Rankin Inlet, Nunavut, Canada

**Keywords:** Arctic top predator, central place forager, intra-population niche variation, landscape heterogeneity, peregrine falcon, reproductive success

## Abstract

While intra-population variability in resource use is ubiquitous, little is known of how this measure of niche diversity varies in space and its role in population dynamics. Here we examined how heterogeneous breeding environments can structure intra-population niche variation in both resource use and reproductive output. We investigated intra-population niche variation in the Arctic tundra ecosystem, studying peregrine falcon (*Falco peregrinus tundrius,* White) breeding within a terrestrial-marine gradient near Rankin Inlet, Nunavut, Canada. Using stable isotope analysis, we found that intra-population niches varied at the individual level; we examined within-nest and among-nest variation, though only the latter varied along the terrestrial-marine gradient (i.e., increased among-nest variability among birds nesting within the marine environment, indicating higher degree of specialization). Terrestrial prey species (small herbivores and insectivores) were consumed by virtually all falcons. Falcons nesting within the marine environment made use of marine prey (sea birds), but depended heavily on terrestrial prey (up to 90% of the diet). Using 28-years of peregrine falcon nesting data, we found a positive relationship between the proportion of terrestrial habitat surrounding nest sites and annual nestling production, but no relationship with the likelihood of successfully rearing at least one nestling reaching 25 days old. Annually, successful inland breeders raised 0.47 more young on average compared to offshore breeders, which yields potential fitness consequences for this long-living species. The analyses of niche and reproductive success suggest a potential breeding cost for accessing distant terrestrial prey, perhaps due to additional traveling costs, for those individuals with marine nest site locations. Our study indicates how landscape heterogeneity can generate proximate (niche variation) and ultimate (reproduction) consequences on a population of generalist predator. We also show that within-individual and among-individual variation are not mutually exclusive, but can simultaneously arise and structure intra-population niche variation.

## Introduction

Intra-population variability in resource use is ubiquitous and several empirical studies identified among-individual niche variation as a main driver (reviewed in Bolnick et al. 2003). A recent study further showed that decoupled variation in population and individual niches could also arise via increased within-individual variation under conditions of ecological release from competition (Bolnick et al. 2010). Prior studies have highlighted the tendency for top predators to exhibit niche variation, and also their sensitivity to variation in prey abundance (Urton and Hobson 2005; Matich et al. 2011; Dalerum et al. 2012). To help cope with uncertainty, predator species commonly use a cocktail of resources coming from various ecosystems, a factor contributing to niche expansion (Ben-David et al. 1998; Rose and Polis 1998; Restani et al. 2000; Tarroux et al. 2012). Along with this resource subsidization, several factors (biological, ecological or environmental) can interact to shape niche variation (Bolnick et al. 2003; Svanback and Bolnick 2007; Tinker et al. 2008). For example, Darimont et al. (2009) demonstrated that grey wolves (*Canis lupus* Linnaeus) inhabiting different landscapes in a large-scale coastal gradient had increased their niche width through both a surge in consumption of marine-based subsidies and release from inter-specific competition.

Beyond niche variation and its causal mechanisms, few studies have addressed the links between individual niche variation and demographic processes such as reproductive performance (but see Annett and Pierotti 1999; Golet et al. 2000; Votier et al. 2004). Recent work of Giroux et al. (2012) provided evidence that differences in resource abundance within a heterogeneous landscape can influence both resource use and reproduction probability in a generalist predator, the arctic fox (*Vulpes lagopus* Linnaeus). Their study pointed out the importance of fine scale investigation using both spatial and behavioural perspectives to understand consumers' variation in trophic niche and reproductive output (Giroux et al. 2012).

**Figure 1:**
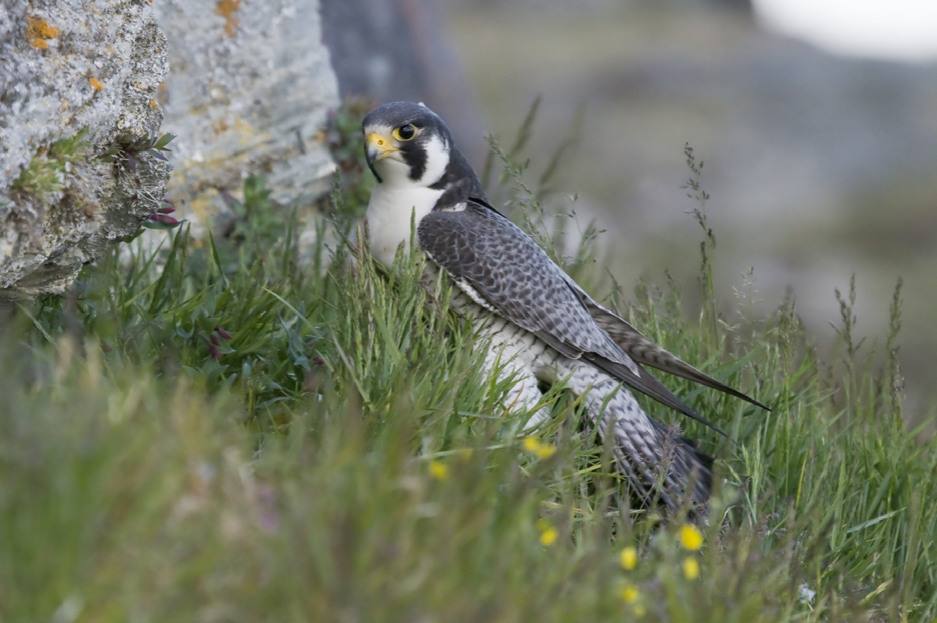
Male peregrine falcon (*Falco peregrinus tundrius*) standing next to his nest on the mainland at the beginning of the nestling rearing period (July) in summer 2008 near Rankin Inlet, Nunavut, Canada.

On the northwestern end of Hudson Bay near the community of Rankin Inlet (Nunavut, Canada) an extensive monitoring program of a top predator, the peregrine falcon *Falco peregrinus tundrius* White, has been ongoing since 1982 (Court et al. 1988; Franke et al. 2011) (Fig. 1). Initially launched to study contamination levels of dichloro-diphenyl-trichloroethane (DTT) after the peregrine falcon was listed as a threatened species under the Canadian *Species at Risk Act* (Cooper and Beauchesne 2007), this program provides long-term monitoring of breeding success and short-term sampling of resource use, an avian parallel to what was done on arctic foxes by Giroux et al. (2012). The multi-species diet of the arctic-breeding peregrine falcon (Cade 1960; Hunter et al. 1988; Rosenfield et al. 1995) makes it an ideal study species for examining niche variation. Compared with their southern counterparts which rely on a bird prey base (Ratcliffe 1980; White et al. 2008), peregrine falcons nesting in the Arctic are regularly observed using mammalian prey species (lemmings -*Lemmus trimucronatus* and *Dicrostonyx groenlandicus* Traill- and ground squirrel *Spermophilus paryii* Richardson) and this behaviour may bear consequences on demographic processes (e.g*.,* lemmings spp.; Court et al. 1988; Bradley and Oliphant 1991 in the Canadian Arctic, Lecomte, A. Sokolov and V. Sokolov, pers. comm., in the Russian Arctic).

**Figure 2:**
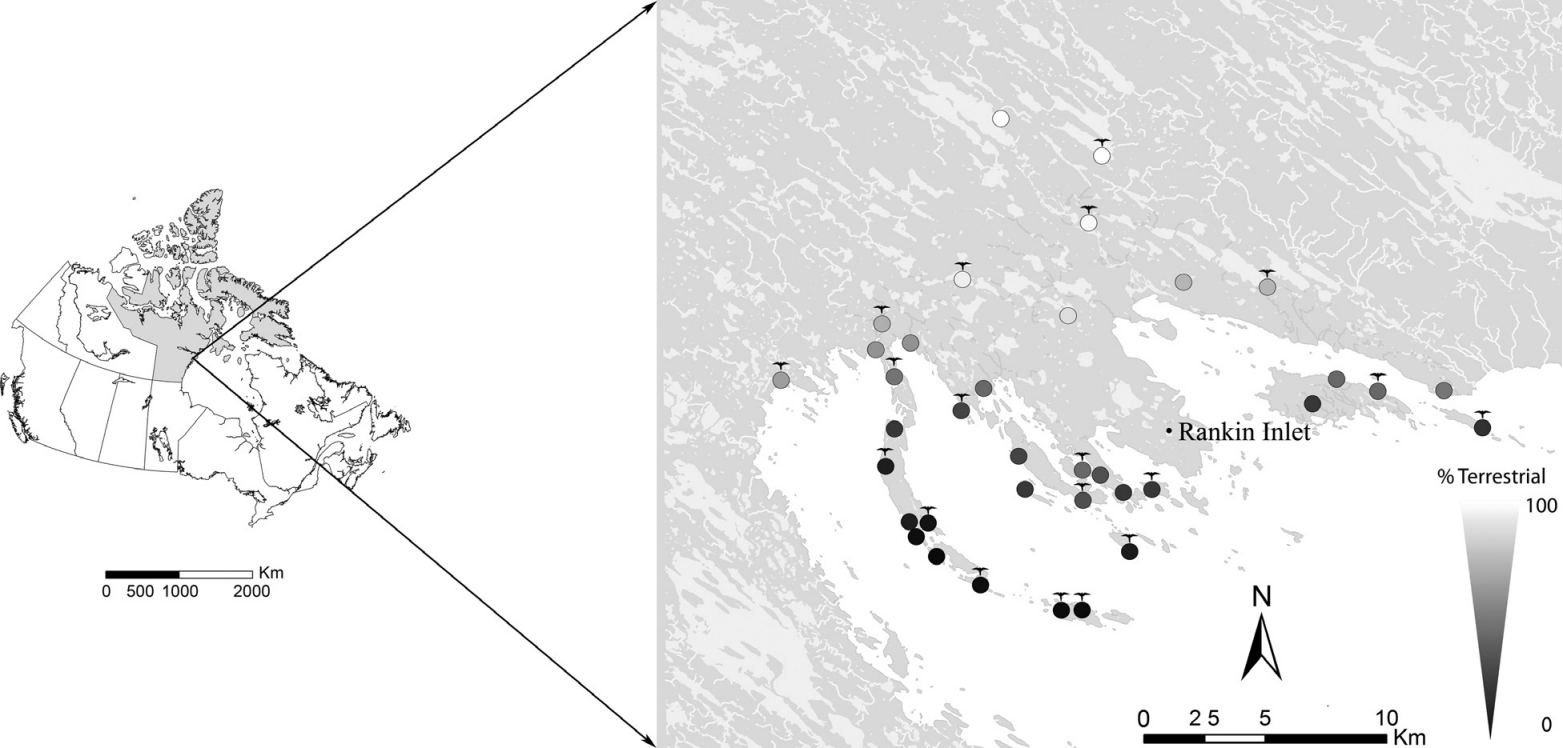
Location of the peregrine falcon study area near Rankin Inlet, Nunavut, Canada. The enlargement shows the study area with the mainland (shaded gray) and marine (blank) habitats. Circles represent breeding sites (*n* = 36; 1982–1999, 2002–2010) and bird symbols above circles highlight falcon breeding sites (*n* = 19) that were successful in raising offspring up to 25 days old in 2008. The intensity of gray shading within circles is proportional to the amount of terrestrial habitat within the falcon's pseudo home range (PHR), from black (0%) to white (100%; see Materials and Methods for details).

Our study was conducted at the junction of the tundra and marine ecosystems, with a mosaic of mixed terrestrial and marine habitat. During the breeding season, the peregrine falcon population is distributed along an environmental gradient (<20 linear kilometers), which provides a unique opportunity to gain insight into how ecological patterns (i.e., intra-population niche variation and reproduction) change relative to environmental factors (Keddy 1991). The landscape heterogeneity generated among-individual variation in the type of the habitat available around nest sites, which varied from terrestrially dominated rock outcrops, to cliffs on small islands surrounded by sea (Figs. 1, 2). Because peregrine falcons stay near their nest and behave as central place foragers (Orians and Pearson 1979), foraging costs for breeding adults may be proportional to the distance between breeding and foraging sites. As a consequence, resource use and reproductive success in peregrine falcons could vary according to the type of habitat (i.e., terrestrial *versus* marine) locally available around nest sites (hereafter; heterogeneity of the breeding environment), if individuals experience different foraging costs along the environmental gradient.

The main objectives of our study were to determine the influences of the heterogeneity of the breeding environment (1) on intra-population niche variation (i.e., within-individual variation and among-individual niche variation) and (2) on individual resource use and annual reproductive success of a generalist predator. We predicted that the use of terrestrial prey would be the greatest by peregrine falcons nesting within or near to the mainland habitat and would decrease with increasing distance between nest sites and the mainland. We also predicted that the annual reproductive success of peregrine falcons would be inversely proportional to the availability of terrestrial habitat within their nest neighbourhood. We examined our predictions using stable isotope sampling (Layman et al. 2012) and long-term monitoring of breeding performance.

## Materials and Methods

### Study area

Our work was conducted near the community of Rankin Inlet, on the western coast of Hudson Bay, Nunavut, Canada (62°49′N, 92°05′W; Fig. 2). Our study area encompassed 349 km^2^, shared between the terrestrial tundra (mainland) and marine ecosystems. The inland tundra is composed of low rolling hills with mesic tundra interspersed with wetlands, while the marine ecosystem includes numerous inner and outer islands covered by mesic tundra (Fig. 2). Rocky outcrops and cliffs are prominent features and attract breeding raptors. Outcrops large enough for falcon nests occur up to 9 km inland and on islands up to 4 km from the coastline. Average peregrine falcon nest site density in the study area is one nest per 8.73 km^2^ (A. Franke, M. Bradley, G. S. Court, C. Hotson, N. Lecomte and M. Setterington, unpubl. data). The terrestrial fauna found in the study area is typical of the low arctic tundra (Callaghan et al. 2004); see Appendix 1.

### Study design: quantifying the heterogeneity of the breeding environment

To assess whether the heterogeneity of the breeding environment influenced the diet of nestling falcons and adult reproductive success for nestling rearing falcons (mid July to late August), we first calculated a pseudo home range for each pair of breeding falcon by buffering each nest with a 5 km radius circle (Hunter et al. 1988; Byholm et al. 2007). Five kilometers was selected because it is within the typical range of foraging distance for breeding peregrine falcon observed in other studies (A. Franke, M. Prostor, V. L'Hérault and J. Bety, unpubl. data). Secondly, we characterized these pseudo home ranges by calculating the proportion of terrestrial (mainland plus islands) to marine (sea water) habitat present within the 5 km buffer using ArcGIS 9.2 software (ESRI, Redlands, CA). We then used the pseudo home range of the nests distributed along the terrestrial-marine continuum and isotopic ratios of nestlings to assess relationships between habitat, diet heterogeneity and the production of young, as a measure of adult reproductive success.

### Peregrine falcon monitoring and prey sampling

During the breeding seasons from 1982 to 2010 (except 2000 and 2001; no data available), we monitored peregrine falcon nests across the study area and recorded the number of young produced. During the 28 years of monitoring, all active falcon nests within the study area were visited at least once to count and band nestlings when they were approximately 25 days old, prior to their fledging age (∼35–40 days old; Ratcliffe 1980). We classified a nest as successful if at least one nestling reached 25 days old (banding age). Additionally between 1982 to 1995, and 2008 to 2010, we systematically recorded breeding activities from laying to fledging; this provided a more detailed description of breeding parameters including clutch size and number of young hatched.

During the summer of 2008, we monitored nestling diet at all active nests, starting 14 days after hatch date (mid-July) until nest departure around mid August (Appendix 3). To capture diet variation over the course of this nestling period we used stable isotope ratios (δ^13^C and δ^15^N) of blood plasma to track nutrients consumed and assimilated over a very fine temporal window (weekly) (Hobson and Clark 1993) (Appendix 3). We collected blood samples (1 mL) from the ulnar vein every 5–12 days (7.7 ± 1.6 standard deviation [SD] days) from 50 nestlings at 20 nests (1.8 ± 1.3 SD nestlings per breeding nest). We obtained an average of 2.5 ± 0.7 SD (range 1–3) blood measurements per nestling throughout the nestling period. Blood samples were sealed, stored in heparin in the field and centrifuged within 8 h of collection to separate plasma from red blood cells. All samples were stored frozen until further analysis (see below).

During the falcon nestling period, we opportunistically collected 86 specimens from 13 prey species in the study area (Appendix 2). The selection of prey items was based on previous years' observations made during the nestling period via analyses of prey remains (Court et al. 1988) and from information collected by scouting cameras placed at nests (*n* = 5 evenly distributed within the environmental gradient; unpubl. data). Muscle samples were extracted from prey items and stored in 70% ethanol prior to analyses (Ehrich et al. 2010).

### Stable isotope analyses

To reconstruct the diet of the peregrine falcon nestlings, we first measured the δ^13^C and δ^15^N of nestlings' plasma and prey muscle samples. After initial preparation including lipid extraction of prey samples (see details in Appendix 4), we determined isotope signatures using a continuous flow Finnigan Mat Delta Plus isotope ratio mass spectrometer at Stable Isotopes in Nature Laboratory (SINLAB), University of New Brunswick, Canada. Stable isotope ratios are expressed as parts per thousand (‰) deviations from standards, namely Pee Dee Belemnite for C and atmospheric air for N (Appendix 4).

### Data analyses

All analyses were run using packages written for the R 2.12 software (R Development Core Team 2012).

#### Intra-population niche variation

To investigate whether the heterogeneity of the breeding environment influenced within-individual and among-individual niche variation in falcons, we correlated the proportion of terrestrial habitat within the falcon's pseudo home range with two metrics, (1) the “mean within-nest distance” and (2) the “mean among-nest distance” calculated from the relative positioning of nestlings in the δ^13^C-δ^15^N bi-plot (Appendix 5) (Layman et al. 2007; Turner et al. 2010). We measured niche variation at the scale of the nest. This is a proxy of individual niche variation because the siblings from a single nest are all fed by the same individuals (parents) (Ratcliffe 1980).

We refer to the “niche” and “ecological specialization” concepts following the framework developed by Poisot et al. (2011). Individuals with higher degree of specialization are those for which the niche is substantially narrower than the population niche (Bolnick et al. 2003). The mean within-nest distance is positively correlated to individual generalization (greater within-nest distance reflects a larger individual niche width) and mean among-nest distance is positively correlated to individual specialization (greater among-nest distance reflects individual spread apart within the population niche). Because the metrics were calculated from data collected during the rearing season using short-term diet trackers, they represent niche variation for this particular time frame (from mid-July to mid-August).

We calculated the two metrics by first computing the centroid of each nest (a point representing the average position of siblings in the isotopic space) and then calculating the Euclidean distance between this nest centroid and the relative position of each sibling (mean within-nest distance) or the relative position of the other nest centroids (mean among-nest distance; see Appendix 5 for an example) (Layman et al. 2007). In order to track the differences in among-nest variation along the environmental gradient (terrestrial to marine), we measured the Euclidean distances between a nest and the five nests with the closest values for the variable “proportion of terrestrial habitat within the falcon pseudo home range” (habitat neighbours). Calculating the mean distance among five neighbouring nests, rather than more or less, balanced both a minimal level of replication and discrimination of the among-nest variation along the environmental gradient. For instance, the mean distance calculated out of 4, or six neighbours, would lead to either insufficient replication or low discrimination; see Appendix 6 for a sensibility analysis using linear regression models.

#### Diet

We used Stable Isotope Analysis in R (SIAR; Parnell et al. 2010) to reconstruct the diet of peregrine falcon nestlings. Our aim was to investigate the relative importance of terrestrial versus marine prey source for peregrine falcon nestlings across the terrestrial/marine continuum. Isotopic signatures of peregrine falcon nestlings sampled in 2008 were then modeled with respect to the nest they belonged to (*n* = 19) so that the reconstructed diet could be compared according to the variable “proportion of terrestrial habitat within falcon's pseudo home range.” Because we worked with a large number of potential sources (13 prey species, Appendix 2) and only two isotopic tracers, we used a multivariate analysis of variance (MANOVA) (tested for the two assumptions of normality of the distribution and homogeneity of the variance) to determine whether isotopic ratios of different prey sources were sufficiently clustered to be pooled together (Phillips and Koch 2002). We then pooled prey species to three distinct types: terrestrial herbivores (three species), terrestrial insectivores (seven species) and marine birds (three species) (MANOVA, Wilks' Lambda: *F*_2,83_ = 149.9, *P* < 0.001, *n* = 86; Appendix 2). We corrected isotopic ratios of peregrine falcon nestlings for isotopic discrimination (i.e*.,* difference between the isotope signatures of the diet and the tissue of the consumers) using estimates developed for whole blood of adult falcons fed a simple diet composed of quail (δ^13^C = 0.2 ± 0.01 SD, δ^15^N = 3.3 ± 0.4 SD; Hobson and Clark 1992). Although discrimination factors may vary between plasma and blood cells, and between young and adult (Lecomte et al. 2011), using an average for the whole blood is a conservative approach. We also take into account the concentration dependence of C and N in tissues. We ran SIAR using the following parameters: iterations = 1,00,000, burnin = 100,000, thinby = 10, and flat priors (Parnell et al. 2010).

#### Reproductive success

To investigate whether the heterogeneity of the breeding environment influenced reproductive success in the population, we compared the number of young per nest at the time of nestling banding (∼25 days old) over the 28 years of monitoring (1982–1999, 2002–2010) to two fixed predictors: the proportion of terrestrial habitat within falcon's pseudo home range and the year. We integrated the nest site identity (nest site use varied over the duration of study) as a random variable in the linear mixed-effect models. The number of nestlings to reach banding age was the most consistently recorded variable across all years of the study. We lack details on nestling mortality therefore, only successful pairs (i.e*.,* those pairs that raised at least one young to 25 days old and were detected during yearly nest visits) were included when modeling reproductive success. Additionally, we were able to model nest success (no young vs. 1 or more young produced) for 17 years (1982–1995; 2008–2010). We used a binomial distribution within a linear mixed-effect model to address the contribution of the proportion of terrestrial habitat within falcon's pseudo home range (fixed), year (fixed), and nest identity (random) to variation in nest success. To ensure that the effect of the heterogeneity of the breeding environment was not related to phenomena occurring outside of the nestling period, clutch size together with the number of young hatched were also used as response variables in linear mixed-effect models. For all linear models used in our study, we verified the assumption of linearity and homoscedasticity.

## Results

### Heterogeneity of the breeding environment

The proportion of terrestrial habitat within a falcon's pseudo home range was highly variable (5–100%) and averaged 45 ± 29% SD (*n* = 36 nests, Fig. 2). This variability was similar within the subset of nests sampled in 2008 for our isotope analysis (45 ± 38% SD, *n* = 19 nests, Fig. 2). This suggests that the nest site distribution in 2008 was comparable that of the 36 nest sites monitored over 28 years (Fig. 2).

### Intra-population niche variation

**Figure 3:**
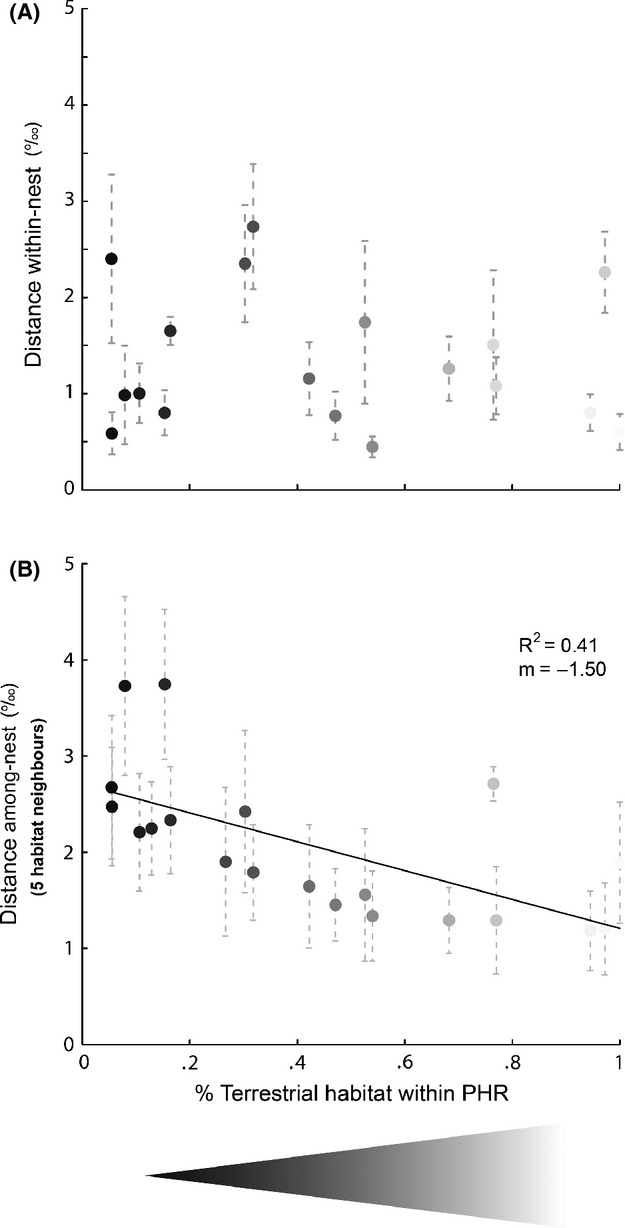
Mean within-nest distance (A) and mean among-nest distance (to five habitat neighbours) (B) relative to the proportion of terrestrial habitat within the pseudo home range (PHR) for peregrine falcon nestlings sampled up to three times during nestling period 2008 near Rankin Inlet, Nunavut, Canada. Circles illustrate the mean nest distances and bars represent standard deviation. The intensity of gray shading within circles is proportional to the amount of terrestrial habitat within the falcon's pseudo home range, from all black (0%) to all white (100%).

**Figure 4:**
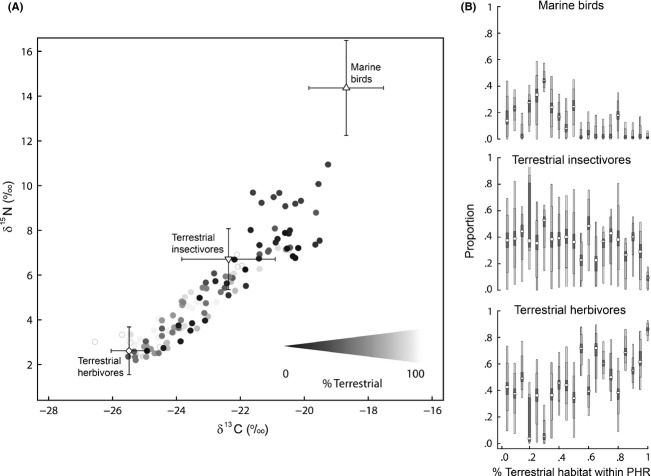
Influence of the proportion of terrestrial habitat within the falcon's pseudo home range (PHR) on (A) the isotopic signature of peregrine falcon nestlings and (B) the relative contribution of their potential prey species to their diet for summer 2008 near Rankin Inlet, Nunavut, Canada. Left panel (A): circles represent young falcons sampled up to three times during the nestling period, and arrows represent their potential prey species gathered in three clusters (mean ± standard deviation). The intensity of gray shading within each circle is proportional to the amount of terrestrial habitat within the falcon's pseudo home range, from all black (0%) to all white (100%). Data are corrected for isotopic discrimination for δ^13^C and δ^15^N. Right panel (B): Stable Isotope Analysis in R (SIAR) output distributions of the relative contribution of marine birds, terrestrial insectivores and terrestrial herbivores in the reconstructed diet of peregrine falcon nestlings. Boxplot showed the 5, 25, 75 and 95 credible intervals (white marks, dark grey, light gray and white boxes, respectively) of the SIAR posterior probability distributions.

At the population level, the value of the mean isotopic within-nest distances was 1.3 ± 0.5‰ SD (ranging from 0.4 ± 0.2‰ SD to 2.7 ± 1.3‰ SD). A quarter of the nests (five nests) showed relatively high and variable values for their mean within-nest distance metric, whereas the majority of the nests showed lower and consistent values (Fig. 4A). Mean within-nest distance variation was independent of the proportion of terrestrial habitat within falcon's pseudo home range (linear regression model; *m* = −0.23 ± 0.52 standard error [SE], *n* = 17, Fig. 3A). Although possible diet generalists may exist in the population (increased within-nest variability), the majority of individuals were likely using a limited set of prey mixture over the rearing season. The mean isotopic among-nest distances (to five habitat neighbours) were higher (ranging from 1.3 ± 0.4‰ SD to 3.7 ± 1.0‰ SD) than the mean within-nest distances (1.3 ± 0.5‰ SD). In addition, the variation in mean isotopic among-nest distances was negatively related to the proportion of terrestrial habitat within the falcon's pseudo home range (linear regression model; *m* = −1.50 ± 0.42 SE, *n* = 19, Fig. 3B). This suggests individual's diet specialization (increased among-nest variability) was a driving mechanism structuring niche variation within the marine-dominated environment.

### Diet

We recorded a steep increase in δ^13^C and δ^15^N ratios from terrestrial prey species compared to marine prey species (ranging −26.0 to −17.4 in δ^13^C and 1.4–16.7 in δ^15^N: Appendix 2 and Fig. 4A). We found a large variation in stable isotope ratios of falcon nestlings raised along the terrestrial-marine continuum (ranging −26.6 to −19.3 in δ^13^C and 2.2–10.9 in δ^15^N: Fig. 4A). The contribution of terrestrial herbivores prey source in peregrine falcon nestlings diet notably increased with increased proportion of terrestrial habitat within falcon's pseudo home range, ranging from a CI 95% equaled to [0%, 30%] in an offshore nest site to a CI 95% [78%, 95%] in a mainland nest site (Fig. 4B). However, the contribution of marine birds prey source in peregrine falcon nestlings diet notably decreased with increased proportion of terrestrial habitat within falcon's pseudo home range, ranging from a CI 95% equaled to [36%, 57%] in an offshore nest site to a CI 95% [0%, 9%] in a mainland nest site (Fig. 4B). Finally, the contribution of terrestrial insectivores prey source in peregrine falcon nestlings diet also varied among nest sites but to a lesser extent than for marine and terrestrial prey, ranging from a CI 95% equaled to [15%, 95%] in an offshore nest site to a CI 95% [0%, 18%] in a mainland site (Fig. 4B). The overall pattern indicates a predominance of terrestrial prey source regardless of nest positions within the terrestrial/marine landscape.

### Reproductive success

During the 28 years of monitoring, 11.9 ± 4.5 SD peregrine falcon nests had at least one 25 day-old young (range: 1–20 nests/year; *n* = 36 nest sites). The population averaged 28.7 ± 14.2 SD young annually (range: 3–61), with an average of 2.4 ± 0.4 SD young produced per successful nest (range: 1–4).

**Figure 5:**
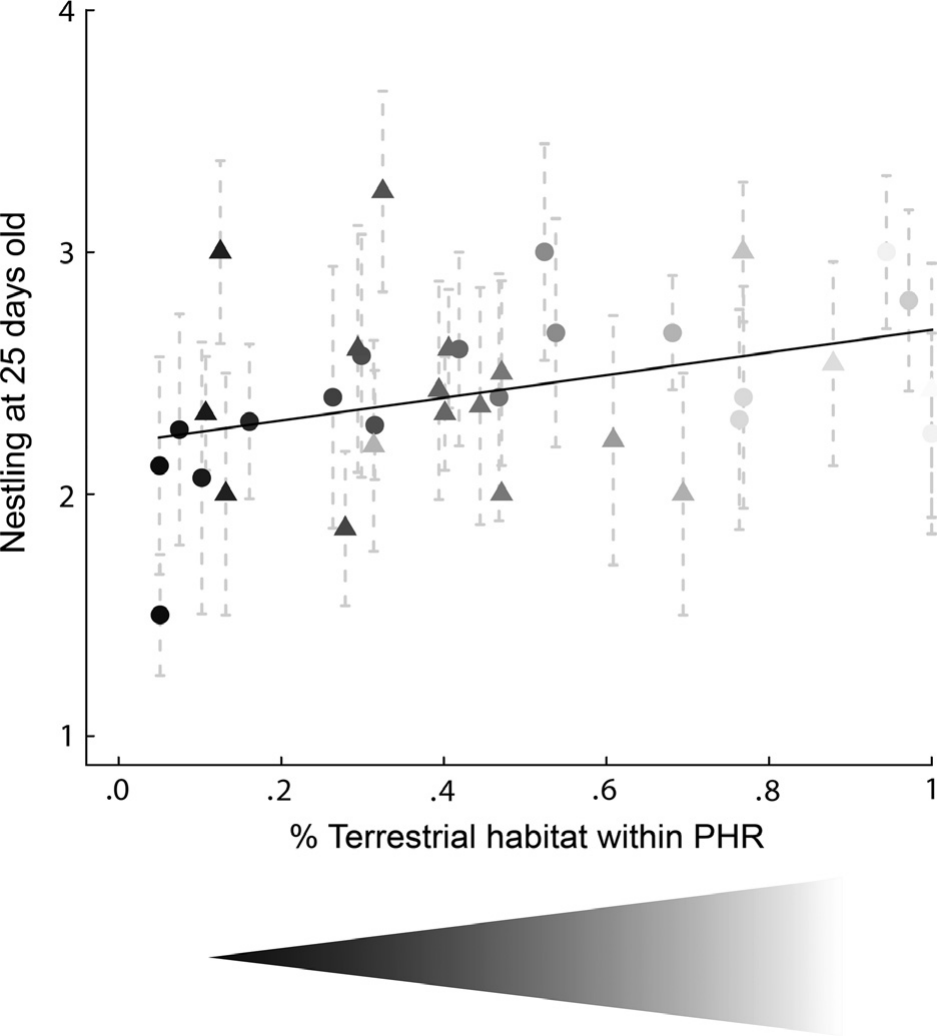
Influence of the proportion of terrestrial habitat within the falcon's pseudo home range (PHR) on the mean number of fledglings produced near Rankin Inlet, Nunavut, Canada (1982–1999, 2002–2010). Dots represent the average number of nestlings produced per nest (*n* = 36) and arrows show standard error. The line indicates fitted values for illustrative purposes only. Triangles highlight the distribution of the nests sampled in 2008 for stable isotope work. The intensity of gray levels are proportional to amount of terrestrial habitat within the falcon's pseudo home range, from all black (0%) to all white (100%).

The number of young produced per successful nest was positively related to the proportion of terrestrial habitat within the falcon's pseudo home range (number of young produced increased by 0.47 from a marine-dominated pseudo home range to a terrestrial-dominated one [*m* = 0.47 ± 0.17 SE, *n* = 36]), and decreased over time (number of young produced decreased by 0.14 per 10 years [*m* = −0.014 ± 0.006 SE, *n* = 28]) (Table 1A and Fig. 5). In contrast, peregrine falcon nest success (no young vs. 1 or more young produced) was not related to the proportion of terrestrial habitat within the pseudo home range (*m* = 0.17 ± 0.52 SE, *n* = 36), (Table 1B). Similarly, clutch size (*m* = 0.04 ± 0.16 SE, *n* = 36) and number of eggs hatched (*m* = 0.25 ± 0.33 SE, *n* = 36) were not related to the proportion of terrestrial habitat within falcon's pseudo home range, (Table 1A). This suggests that the effect of the heterogeneity of the breeding environment on the number of young produced was associated with events occurring during the nestling period.

**Table 1:** Summary of the linear mixed-effect models accounting for the effect of the proportion of terrestrial habitat within the peregrine falcon's pseudo home range (PHR) and year (Year) on the number of young produced (1982–1999 and 2002–2010) and on the nest success, number of young hatched and clutch size (1982–1995 and 2008–2010). Reported within the linear mixed-effect model are (A) a continuous distribution and (B) a binomial distribution. Models accounted for the effect of *nest identity* as a random variable but not presented here

Response	Predictor	*b*	Standard error	df	*t*	*P*
(A)						
Young produced	PHR	0.47	0.17	34	2.69	0.01**
	Year	−0.014	0.006	283	2.37	0.02*
Young hatch	PHR	0.25	0.33	34	0.75	0.46 ns
	Year	0.011	0.009	290	1.22	0.22 ns
Clutch size	PHR	0.04	0.16	34	0.31	0.76 ns
	Year	0.007	0.005	290	1.53	0.13 ns
(B)						
Nest success	PHR	0.17	0.52	34	0.32	0.75 ns
	Year	−0.027	0.015	330	1.80	0.072 ns

Data collected near Rankin Inlet, Nunavut, Canada.

**, *, and ns represent *P* < 0.01, *P* < 0.05, and *P* > 0.05 (non-significant), respectively.

## Discussion

It has been known that landscape heterogeneity can influence ecological processes such as intra-population niche variation, (e.g., Darimont et al. 2009), but little is known about the underlying mechanisms, and their demographic consequences on populations. By combining analyses of an individual's isotope niche with long-term monitoring of reproduction, our study shows how landscape heterogeneity (terrestrial/marine gradient) can influence a generalist predator population: the proportion of terrestrial prey source within a peregrine falcon nestling diet and the brood size decrease with increasing nest site distance to the mainland. Breeding within the mainland habitat potentially yields a fitness advantage with this long-living species.

Here we present robust results from (1) the monitoring and sampling of all hatchlings in active nests for isotopes during 2008, and (2) the integration of all nests present during 28 years of population monitoring (nest detection probability was high due to high nest site fidelity; Franke et al., unpubl. data). Moreover, the distribution of nest sites in 2008 across the habitat gradient was representative of the distribution of all used nest sites (*n* = 36; Fig. 2) recorded during the 28 years and allows for extrapolation of the niche/landscape relationship for a multi-year perspective (Figs. 2, 5). However, our understanding of the effect of landscape heterogeneity on resource use could be furthered by calculating the proportion of terrestrial habitat within actual foraging area in place of our pseudo home range.

### Landscape heterogeneity effects on intra-population niche variation

Bolnick et al. (2003) have demonstrated that many apparent generalist species can be in fact composed of a range of ecologically variable individual specialists. Our results from isotope analyses indicated that niche variation within the peregrine falcon population arose from individuals with variable degree of generalization (high intra-nest variation) and specialization (high among-nest variation) in their prey use. These findings show that (1) peregrine falcons, considered as generalist predators, can actually exhibit a higher-than-anticipated degree of dietary specialization during the breeding season and (2) individual specialization and generalization are not mutually exclusive phenomena but can simultaneously arise and structure intra-population niche variation (see also Tinker et al. 2008). Interestingly, we found a higher degree of specialization with individuals nesting offshore than with those individuals nesting in terrestrial-dominated habitats, which is in contrast to conclusions drawn in recent studies dealing with similar ecological circumstances (i.e*.,* generalist predator inhabiting a heterogeneous landscape). Darimont et al. (2009) reported that a coastal grey wolf population showed the most specialized individuals (sub-population with the largest trophic niche) under conditions of increased species richness with resource input from the sea (spawning salmon), and Giroux et al. (2012) showed that arctic foxes breeding in the vicinity of a goose colony had increased niche breadth compared to more distant breeders. In our study, peregrine falcons nesting near the mainland (along the coast) had the widest diversity of prey resources in their nesting environment with access to both terrestrial and marine resources, but these individuals did not exhibit the widest niche; in fact, they extensively used terrestrial prey. We address this result with two possible, though not mutually exclusive, explanations.

First, we hypothesize that the peregrine falcon is limited in its ability to use marine resources, which would explain its minimal use by individuals nesting on or near the mainland (with low individual specialization). Under this scenario, the characteristics of marine birds (e.g., capable of diving under water to escape predation) do not complement the predator's traits (e.g., lack of hovering behaviour over the surface of the water, shorter wings, small size, and shorter claws than subspecies specializing on marine birds; Nelson 1990) to allow for a match that yield energetic benefits for the predator (Sih and Christensen 2001; Bolnick et al. 2003; Tinker et al. 2008). The availability of marine prey relative to terrestrial resources may be an important factor influencing profitability (prey use) and needs to be quantified by further studies since no quantitative data are currently available for our study area.

Second, we hypothesize that the abundance of terrestrial resources during 2008 within our study area was high enough to provide food for nesting peregrine falcons on or near the mainland, explaining the low use of marine resources (with low individual specialization and constant isotopic niche). The extensive use of herbivore prey items (rodents) by peregrine falcon nestlings (as shown by isotope modeling), along with measurements (lemming trapping) and observations (high breeding density [25 breeding pairs with the study area] and breeding success of a lemming specialist, the rough-legged hawk, *Buteo lagopus*) during summer 2008, support the hypothesis that lemmings were overabundant in 2008 and, consequently, that the consumption of marine resources may be a minimal estimate for this population over the long term. Other studies have demonstrated that inter-annual variation in preferred resources can modulate the relative contribution of marine resources in the diet of generalist consumers (e.g., Roth 2002). Quantifying the multi-annual variation in lemming abundance, as well as other terrestrial prey species, is necessary to further support our hypothesis and to understand its temporal extent. This could be done by quantifying the contribution of lemming versus marine resources to peregrine falcon diet across different phases of the lemming cycle (peaks and crashes in particular). Despite the low contribution of marine resources to the overall falcon diet, our results show a significant use of marine resources by offshore nesting peregrine falcons. We address these results in the light of the optimal foraging theory that predicts the use of alternate prey (here marine resources) by a consumer to be quite low unless the availability of preferred prey (here terrestrial resources) is decreased (Schoener 1971; Sih and Christensen 2001).

### Landscape heterogeneity effects on reproductive performance

Although our results do not identify the mechanism underlying the decrease in the annual number of young produced by offshore nesting peregrine falcons, analyses of niche and reproductive success suggest a potential breeding cost for accessing distant terrestrial prey. The central place foraging theory (CPFT; an extension of optimal foraging theory; Orians and Pearson 1979) provides some support to this possible explanation. Our studied system fulfils the central premise of the CPFT, as peregrine falcons are bound to a fixed central place (their nest) during the nestling period. Suitable nest sites are available asymmetrically within the study area (most nest sites are available within the marine end of the gradient), yet the apparent preference of peregrine falcons for terrestrial prey (Fig. 4) results in many individuals being unable to choose a central place close to their preferred food distribution (Orians and Pearson 1979). Hence, CPFT expects that peregrine falcons nesting in the most distant location (offshore) relative to foraging areas (mainland) will experience higher traveling costs; this could in turn impact their reproductive output (Orians and Pearson 1979). Interestingly, CPFT also predicts that the most distant nesting peregrine falcons relative to their terrestrial resource would be more likely to integrate locally available resources (i.e*.,* marine resources) to cope with the increased foraging cost, as observed in our study (Fig. 4B). Our results follow such pattern by using the angle of the niche theory to understand resource heterogeneity. This merges both niche theory and CPFT into a single framework.

Along with CPFT, recent empirical studies (Hakkarainen et al. 2003; Lambrechts et al. 2004; Byholm et al. 2007; Doligez et al. 2008; Golawski and Meissner 2008) have drawn parallels between habitat quality (resource availability), food delivery rates (energy intake for nestlings) and reproductive performance. For example, Byholm and Kekkonen (2008) experimentally demonstrated that small-scale variation in habitat quality, along with food availability, could influence demographic patterns in “habitat-sensitive” avian top predator (goshawk *Accipiter gentilis* Linnaeus). Assuming increased traveling costs for peregrine falcons breeding offshore (as suggested by our results), how this could resulted in a decreased food provisioning would require the quantification of the delivery rates at nests and the correlation of these with the proportion of terrestrial habitat with the peregrine falcons' home range.

Since our study was not experimental, we cannot exclude the possibility that the observed relationship between reproductive response (and resource use) and landscape heterogeneity may be linked to other mechanisms. First, individual quality has been shown to influence reproductive performance (Carrete et al. 2006); in our study area individual quality could be confounded with landscape heterogeneity influences, as peregrine follow a despotic distribution where high quality individuals often monopolize high quality habitat (Sergio et al. 2009). However, as we detected no relationship between the proportion of terrestrial habitat within falcons' pseudo home range and clutch size (a proxy of individual quality; Sydeman et al. 1991), we argue that variation in individual quality could not solely explain the observed tendency linking landscape heterogeneity, trophic niche, and reproduction. Second, previous studies conducted in the same study area have documented the influence of weather as a major determinant of peregrine falcon nestling survival and reproductive success (Bradley et al. 1997; Anctil 2012). To assess whether weather could create variance in the nestling survival across the landscape gradient, we would need to measure nest sites exposure and weather events at the nest site scale.

### Generalist predators and tundra functioning

When compared to the large contribution of marine energy to the diet (Tarroux et al. 2012) and reproductive success (Roth 2003) of another top arctic predator, the arctic fox, marine inputs for peregrine falcons was only minimal, at least for summer 2008. This highlights the various consequences of marine resources input on generalist predators with contrasting life cycle characteristics. Nevertheless, the contribution of marine resources in the peregrine falcons' diet was significant for individuals with direct access to this resource and remote access to terrestrial resources, boosting breeding output. Assessing the role of marine prey on peregrine falcons' reproduction is necessary to determine how its use by top predators could scale-up to affect demographic processes within the population, and also to assess potential ecosystem consequences (Lecomte et al. 2008; Leroux and Loreau 2008; Killengreen et al. 2011; Giroux et al. 2012).

## Conclusion

Our study shows how heterogeneous breeding environments can generate proximate (variation in resource use) and ultimate (reproduction) consequences on a population of generalist predators during its breeding season. The results we present here contrast with observations made under a similar context (landscape heterogeneity) but at a larger spatial scale and with different species (Darimont et al. 2009; Giroux et al. 2012), thus highlighting the importance of fine-scale investigations of spatial variability and a sound understanding of animal life-history.

## Supporting Information

**Appendix 1.** Species found with the study area, Rankin Inlet, Nunavut.

**Appendix 2.** Stable isotope signatures of prey species found with the study area.

**Appendix 3.** Breeding cycle of peregrine falcons.

**Appendix 4.** Laboratory preparation of falcon and prey tissues for stable isotope analyses.

**Appendix 5.** On the calculation of niche variation metrics.

**Appendix 6.** Results of fitted linear regression.
