# Supplementary material for: Landscape heterogeneity drives intra-population niche variation and reproduction in an arctic top predator
Source: Ecol Evol. 2013 Jul 24;3(9):2867–79. doi: 10.1002/ece3.675 (PMC3790536; doi:10.1002/ece3.675)
Supplement: Supplementary file 1 [file ece30003-2867-SD1.docx]

**Appendix A1: Species found with the study area, Rankin Inlet, Nunavut**

Most land bird species (passerines, shorebird, waterfowl and cranes) found during summer are migratory, except the resident rock ptarmigan *Lagopus muta* Montin, and raven *Corvus corax* L. In the study area, the small rodent community is composed of brown lemming *Lemmus trimucronatus* Richardson, and collared lemming *Dicrostonyx groenlandicus* Traill, as well as ground squirrel *Spermophilus parryii* Richardson, and arctic hare *Lepus arcticus* Ross (Court et al. 1988a, Court et al. 1988b). These terrestrial prey species are widely available on the mainland, yet they can also be found in lower abundances within the tundra habitat covering the offshore islands.

The coastal fauna is primarily composed of migratory marine birds such as long-tailed duck *Clangula hyemalis* L., common eider *Somateria mollissima* L., black guillemot *Cepphus grylle* L.*,* red-breasted merganser *Mergus serrator* L., and herring gulls *Larus argentatus* L. (Court et al. 1988a, Court et al. 1988b). In addition to the peregrine, the area hosts the lemming specialist, rough-legged hawk *Buteo lagopus* Pontoppidan (Poole and Bromley 1988) and the opportunistic arctic fox Vulpes lagopus L. (Roth 2002, Samelius et al. 2007), with rare sightings of gyrfalcon *Falco rusticolus* L. (arctic hare and ptarmigan specialist; Cade 1960, Poole and Bromley 1988), snowy owl *Bubo scandiacus* L. (lemming specialist in summer; Therrien et al. 2011) and short-eared owl *Asio flammeus* Pontoppidan (lemming specialist; Court et al. 1988a). In addition, there are occasional occurrences of wolverine *Gulo gulo* L. (caribou scavenger or predator; Mulders 2001), tundra wolf *Canis lupus* L. (caribou predator; Frame et al. 2008), grizzly bear *Ursus arctos* L. (herbivore and caribou scavenger or predator; Gau et al. 2002), and polar bear *Ursus maritimus* Phipps (marine predator and terrestrial predator in summer; Rockwell et al. 2011).

**Appendix A2: Stable isotope signatures of prey species found within the study area**

Table A2.1. Summary of means and standard deviations (SD) for δ^13^C and δ^15^N values (in **‰)** in blood plasma (predator) and muscle (prey species) sampled near Rankin Inlet, Nunavut, Canada (2008).

|  |  |  |  |  |  |  |  |  |
| --- | --- | --- | --- | --- | --- | --- | --- | --- |
| **Group** | **Species** | ***n*** |  | δ**^13^C (‰)** | **SD** |  | δ**^15^N (‰)** | **SD** |
| *Predator* | Peregrine falcon | 50 |  | -22.8 | 1.76 |  | 8.4 | 2.14 |
|  |  |  |  |  |  |  |  |  |
| Terrestrial | Collared lemming | 10 |  | -26.0 | 0.38 |  | 1.4 | 0.75 |
| *herbivores* | Ground squirrel | 10 |  | -25.1 | 0.36 |  | 2.4 | 0.71 |
|  |  |  |  |  |  |  |  |  |
| Terrestrial | American pipit | 5 |  | -23.0 | 0.73 |  | 6.9 | 0.65 |
| *insectivores* | Horned lark | 10 |  | -23.1 | 0.47 |  | 5.1 | 1.03 |
|  | Lapland longspur | 5 |  | -21.8 | 1.36 |  | 7.0 | 1.12 |
|  | Savannah sparrow | 7 |  | -22.4 | 0.72 |  | 7.9 | 0.54 |
|  | Semi-palmated plover | 6 |  | -21.5 | 1.78 |  | 6.6 | 1.32 |
|  | Semi-palmated sandpiper | 9 |  | -21.6 | 2.22 |  | 7.6 | 0.98 |
|  | Snow bunting | 5 |  | -23.2 | 1.42 |  | 5.9 | 0.54 |
|  |  |  |  |  |  |  |  |  |
| *Marine birds* | Common eider | 6 |  | -17.4 | 0.89 |  | 12.7 | 1.36 |
|  | Guillemot | 9 |  | -19.3 | 0.58 |  | 16.7 | 0.27 |
|  | Long-tailed duck | 2 |  | -19.4 | 0.37 |  | 13.7 | 0.40 |

**Appendix A3: Breeding cycle of peregrine falcons**

Figure A3.1. Breeding cycle of peregrine falcons monitored near Rankin Inlet, Nunavut, Canada. The shaded area represents the nestling period and dotted lines the approximate times of nestling blood sample collections. The approximate dietary period covered by the stable isotopes of the plasma tissue (turnover time) is represented by shaded bar for each sample.

**
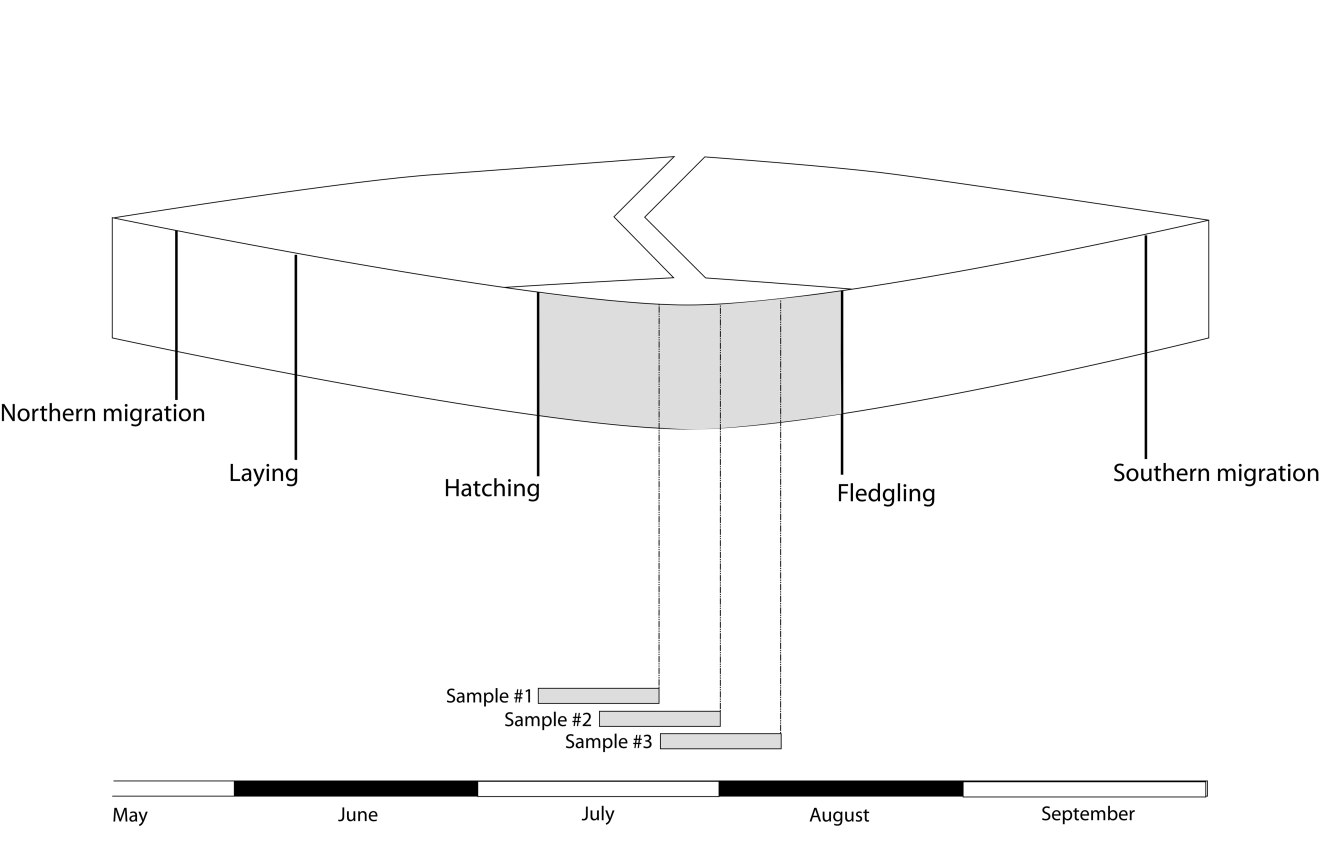
**

**Appendix A4**: **Laboratory preparation of falcon and prey tissues for stable isotope analyses**

*Lipid removal*

Prior to laboratory analyses, we removed lipids from prey muscle with a 2:1 chloroform:methanol solution to control for carbon heterogeneity and lipid differences among different prey muscles (Post et al. 2007). In some studies (e.g., Sweeting et al. 2006), chemical extraction altered δ^15^N to various degrees but in our case, variation in δ^15^N due to lipid extraction was similar to the analytical error (0.14 ± 0.11‰, n = 65 duplicates and 0.11 ± 0.08‰, n = 14, respectively).

Raw and lipid-extracted samples were desiccated and ground to a fine powder using a mortar and pestle. We loaded approximately 0.2 mg of each sample in tin capsules and combusted them in a Carlo Erba NC2500 elemental analyzer. Resultant gases (CO_2_ and N_2_) were delivered via continuous flow to a Finnigan Mat Delta Plus isotope ratio mass spectrometer and analyzed for stable isotopes of carbon and nitrogen at the Stable Isotopes in Nature Laboratory (New Brunswick, Canada).

Stable isotope ratios were expressed in δ-values as parts per thousand (‰) deviations from standards, namely Pee Dee Belemnite for C and atmospheric air for N. following δ^13^C = [(^13^C/^12^C_sample-_^13^C/^12^C_standard_)/^13^C/^12^C_standard_] X 1000 and δ^15^N = [(^15^N/^14^N_sample_-^15^N/^14^N_standard_)/^15^N/^14^N_standard_] X 1000.

Analytical error was estimated to measure accuracy and precision (Jardine and Cunjak 2005). We first estimated accuracy (mean ± SD) with a commercially available standard (acetanilide. Elemental Microanalysis Ltd.): δ^13^C = -27.7 ± 0.12‰ SD and δ^15^N = -2.1 ± 0.12‰ SD (n = 19). Second, precision was measured across runs using an internal smallmouth bass muscle standard (SD): δ^13^C = -23.3 ± 0.04‰ and δ^15^N = 12.5 ± 0.09‰ (n = 16).

Testing the effect of lipid extraction on falcon plasma samples

When reconstructing animal diet with stable isotopes, lipids from tissue samples are usually removed because they are more depleted in δ^13^C compared to protein, therefore reflecting both diet and lipid content (Deniro and Epstein 1978, Ehrich et al. 2011, Tarroux et al. 2011). For instance, Post *et al.* (2007) recommended accounting for lipids (by chemical extraction or mathematical normalization) in lipid-rich tissue as the bias introduced in δ^13^C is positively correlated to lipid concentration. Moreover, accounting for lipid would be important in studies dealing with organisms having variable lipid concentration. Accordingly, lipid removal was an important issue in our study because we reconstructed the diet of falcons based on different prey species (*i.e.,* δ^13^C in falcon samples should be compared to prey after controlling for lipid-induced bias). However, ecologists generally do not remove lipids from plasma, a low-lipid content tissue (Pearson et al. 2003). Hence, to assess whether the plasma samples from falcon nestlings had to be lipid-extracted or not, we conducted a preliminary analysis using a sub-sample of the population (nestlings and adult samples; n = 10 individuals). The mean lipid content of our sub-sample was around 15% (C:N = 4.3 ± 0.24; n = 10 individuals), which is not typical of a low-lipid content tissue (Post *et al.* 2007 considered 10% of lipid as threshold). Indeed, lipid-extracted samples were 1.3 ± 0.14‰ enriched compare to lipid non-extracted samples, which represent a potential bias in our analyses. However, by plotting lipid-extracted samples next to prey ratios, we noticed that lipid removal put falcon samples at the limit of the isotopic range of potential prey species (*i.e.,* the isotopic polygon including standard deviation from prey; Figure 1.1). As the mixing models are sensitive to the positioning of the mixtures relative to the prey polygon (Phillips and Gregg 2003), we considered that the deviation could generate bias and we decided to use the lipid non-extracted samples for the modeling.

Fig. A.4.1. Isotopic ratio in falcons (nestlings and adults; n = 10) for lipid non-extracted (solid dot) and lipid extracted (open circle) plasma samples and their potential prey species gathered in three clusters (mean ± SD) for summer 2008 near Rankin Inlet, Nunavut, Canada. δ^13^C and δ^15^N values were corrected for isotopic discrimination.

**Appendix A5: On the calculation of niche variation metrics**

Figure A5.1. Graphical example of the method used to measure niche variation within the peregrine falcon population. Along the δ^15^N and δ^13^C isotopic bi-plot the a) within-nest variation (a proxy of individual niche variation) is the mean of the Euclidean distances (pale grey line) calculated between each sibling and a centroid representing the mean isotopic position of the nest and b) the among-nest variation (proxy of niche difference among individuals) is the mean of the Euclidean distances (dark grey lines) calculated between the centroid of a nest and the centroids of other nests. In this case, the among-nest variation is calculated for 3 nearest neighbours. This graphic example is based on Layman et al. (2007).


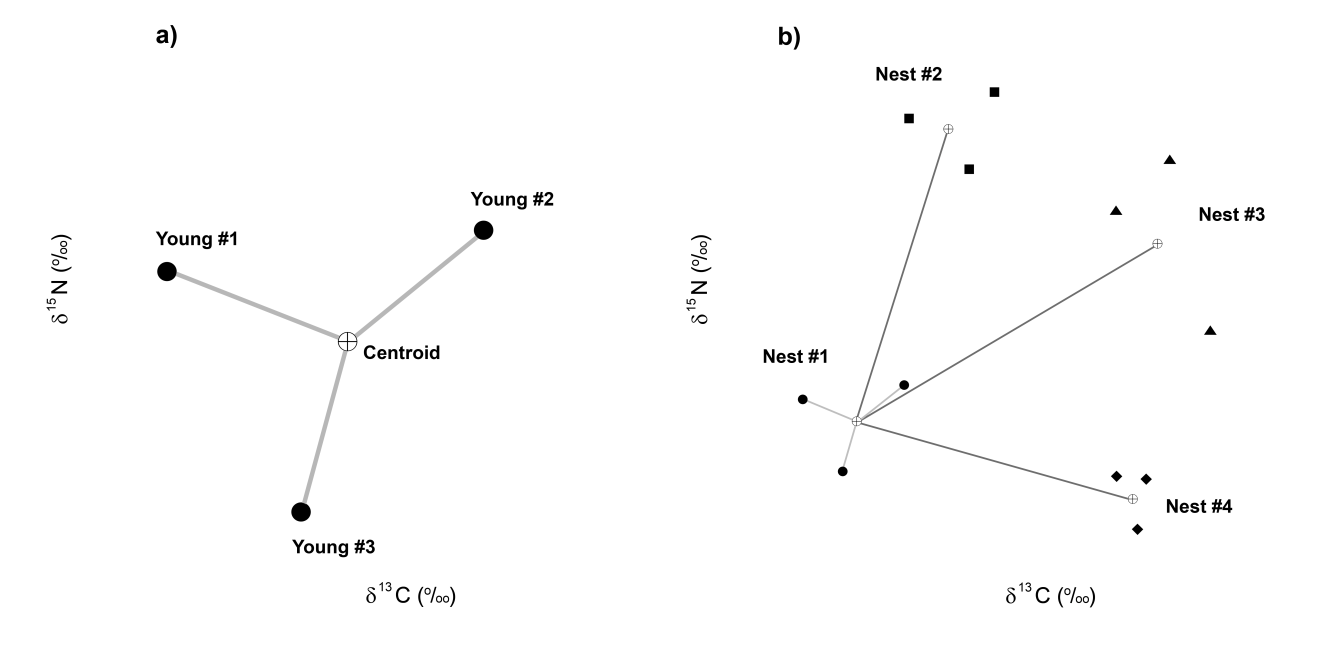


**Appendix A6:** Results of fitted linear regression models

Figure A6.1. Comparison of the mean distance to nearest habitat neighbours with varying number of habitat neighbours (from one to 15) to achieve optimal slope (round) and R^2^ (triangle) scores in fitted linear regression models.

L’Hérault et al. Fig. A6.1.

**Litterature cited**

Cade, T. J. 1960. Ecology of the peregrine and gyrfalcon populations in Alaska. University of California publications in Zoology, 63:151-290. 1982. The falcons of the world, Cornell University press, Ithaca, New York, USA.

Court, G. S., M. Bradley, C. C. Gates, and D. M. Boag. 1988a. The population biology of Peregrine falcons in the Keewatin district of the Northwest territories, Canada. Pages *in* T. J. Cade, J. H. Enderson, C. G. Thelander, and C. M. White, editors. Peregrine falcon population; their management and recovery. The peregrine fund, Boise, Idaho.

Court, G. S., C. C. Gates, and D. A. Boag. 1988b. Natural-History of the Peregrine Falcon in the Keewatin District of the Northwest-Territories. Arctic **41**(1):17-30.

Deniro, M. J. and S. Epstein. 1978. Influence of Diet on Distribution of Carbon Isotopes in Animals. Geochimica et Cosmochimica Acta **42**(5):495-506.

Ehrich, D., A. Tarroux, J. Stien, N. Lecomte, S. Killengreen, D. Berteaux, and N. G. Yoccoz. 2011. Stable isotope analysis: modelling lipid normalization for muscle and eggs from arctic mammals and birds. Methods in Ecology and Evolution **2**(1):66-76.

Frame, P. F., H. D. Cluff, and D. S. Hik. 2008. Wolf reproduction in response to caribou migration and industrial development on the central barrens of mainland Canada. Arctic **61**(2):134-142.

Gau, R. J., R. Case, D. F. Penner, and P. D. McLoughlin. 2002. Feeding patterns of barren-ground grizzly bears in the central Canadian Arctic. Arctic **55**(4):339-344.

Jardine, T. D. and R. A. Cunjak. 2005. Analytical error in stable isotope ecology. Oecologia **144**(4):528-533.

Mulders, R. 2001. Wolverine Ecology, Distribution and Productivity in a Tundra Environment. Final Report. West Kitikmeot / Slave Study Society, Yellowknife, NorthWest territories, Canada, 92p.

Pearson, S. F., D. J. Levey, C. H. Greenberg, and C. M. del Rio. 2003. Effects of elemental composition on the incorporation of dietary nitrogen and carbon isotopic signatures in an omnivorous songbird. Oecologia **135**(4):516-523.

Phillips, D. L. and J. W. Gregg. 2003. Source partitioning using stable isotopes: coping with too many sources. Oecologia **136**(2):261-269.

Poole, K. G. and R. G. Bromley. 1988. Interrelationships within a raptor guild in the central Canadian Arctic. Canadian Journal of Zoology-Revue Canadienne De Zoologie **66**:2275-2282.

Post, D. M., C. A. Layman, D. A. Arrington, G. Takimoto, J. Quattrochi, and C. G. Montana. 2007. Getting to the fat of the matter: models, methods and assumptions for dealing with lipids in stable isotope analyses. Oecologia **152**(1):179-189.

Rockwell, R. F., L. J. Gormezano, and D. N. Koons. 2011. Trophic matches and mismatches: can polar bears reduce the abundance of nesting snow geese in western Hudson Bay? Oikos **120**(5):696-709.

Roth, J. 2002. Temporal variability in arctic fox diet as reflected in stable-carbon isotopes; the importance of sea ice. Oecologia **133**:70-77.

Samelius, G., R. T. Alisauskas, K. A. Hobson, and S. Lariviere. 2007. Prolonging the arctic pulse: long-term exploitation of cached eggs by arctic foxes when lemmings are scarce. Journal of Animal Ecology **76**(5):873-880.

Sweeting, C. J., N. V. C. Polunin, and S. Jennings. 2006. Effects of chemical lipid extraction and arithmetic lipid correction on stable isotope ratios of fish tissues. Rapid Communications in Mass Spectrometry **20**(4):595-601.

Tarroux, A., D. Ehrich, N. Lecomte, T. D. Jardine, J. Bety, and D. Berteaux. 2011. Sensitivity of stable isotope mixing models to variation in isotopic ratios: evaluating consequences of lipid extraction. Methods in Ecology and Evolution **1**(3):231-241.

Therrien, J. F., G. Gauthier, and J. Bety. 2011. An avian terrestrial predator of the Arctic relies on the marine ecosystem during winter. Journal of Avian Biology **42**(4):363-369.
